# What socio-demographic factors support disposable vs. sustainable menstrual choices? Evidence from India’s National Family Health Survey-5

**DOI:** 10.1371/journal.pone.0290350

**Published:** 2023-08-17

**Authors:** Karan Babbar, Supriya Garikipati

**Affiliations:** 1 Jindal Global Business School, OP Jindal Global University, Sonipat, India; 2 School of Politics and International Relations, University College Dublin, Dublin, Ireland; Jawaharlal Nehru University, INDIA

## Abstract

For over a decade, improving menstrual hygiene among poor girls and women in low-and-middle-income-countries has been a prominent global goal. Towards this, governments in the Global South have worked to promote the uptake of disposable sanitary pads. Despite this, we continue to see a high prevalence of period poverty mainly because disposable pads require monthly purchasing that may be burdensome for many women. Not only are pads financially unsustainable but also represent a heavy environmental burden which has kindled an interest in re-usable innovations like menstrual cups that present a sustainable solution. However, there is little understanding of factors that promote the take-up of disposable vs. sustainable products at population levels. In this paper, we draw on India’s National Family Health Survey-5 to understand the socio-demographic determinants of period product usage among girls and women, differentiated by their sustainability quotient. Our findings suggest that awareness of sustainable products and cultural factors are the key driver to promote their use. Women with exposure to menstrual cups either via education or mass media were more likely to use them. Belonging to urban areas and to disadvantaged social categories are other driving factors, at least partly because taboos of vaginal insertion are less of a concern among these groups. These findings suggest that improving the uptake of menstrual cups requires a paradigm shift in menstrual health policies from the promotion of disposable pads alone to spreading awareness of sustainable period choices among women via innovative use of mass media and community networks. Some micro-level evidence of change supports our conclusions.

## Introduction

**Fig 1:**
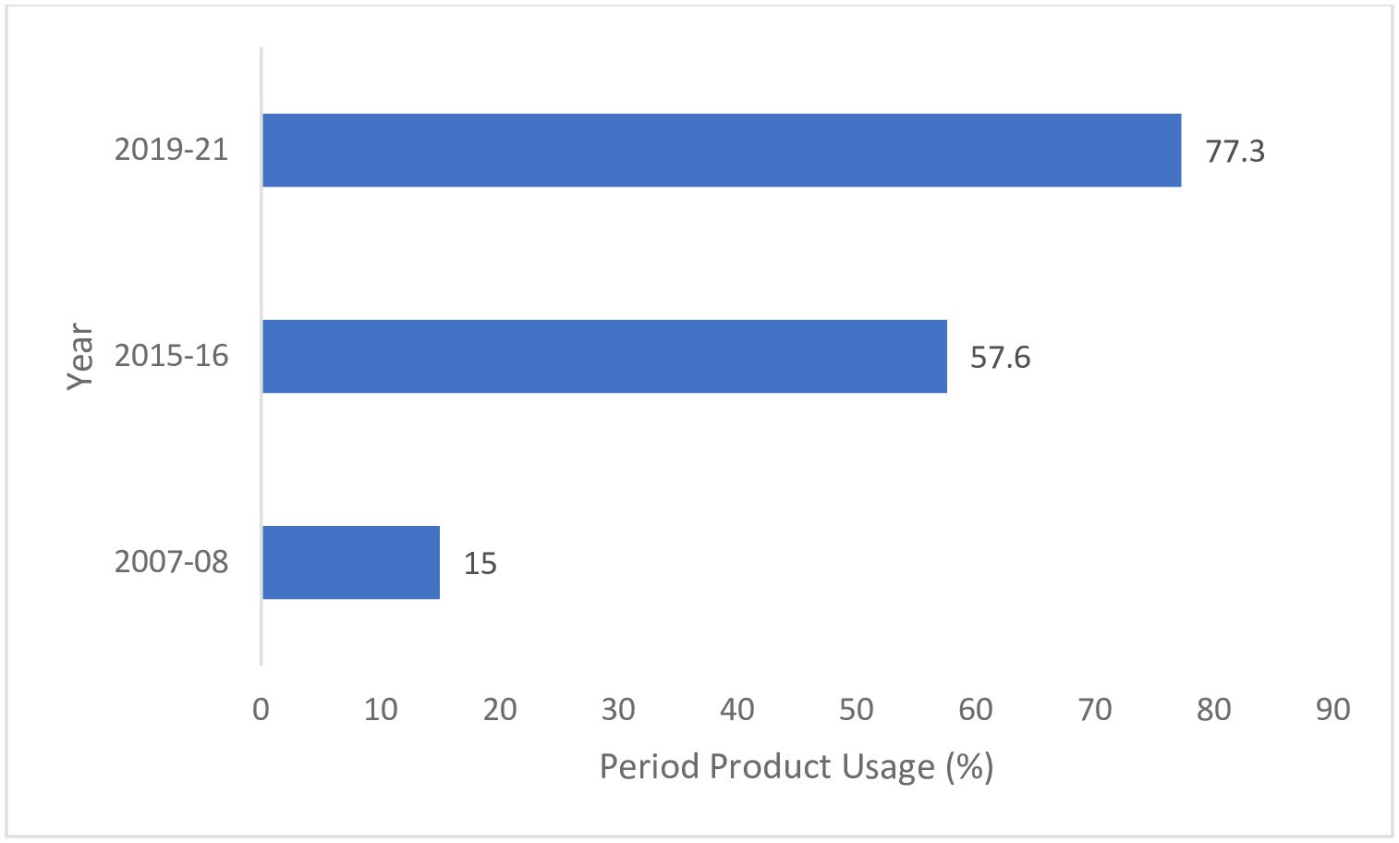
Modern period products usage in India from 2010 to 2022. *Source*: Author estimates using various publicly available datasets [3–5]. *Note*: Data from IIPS and Macro International (NFHS-4 and NFHS-5) is limited to girls and women aged 15–24. Hence, the overall numbers are likely to over-represented period product usage in India. District Level Household Survey (DLHS-3) has data across women aged 15–49 and reports the number of women using sanitary napkins and locally prepared napkins.

High prevalence of period poverty among girls and women in low-and-middle-income- countries (LMICs) is increasingly recognised as a barrier to the attainment of key Sustainable Development Goals like good health, education, and employment [1]. Period poverty is defined as the lack of access to the period products, menstrual health education and Water, Sanitation and Hygiene (WASH) facilities [58]. Several governments in the Global South, including India, initiated policies to promote better menstrual hygiene among their populations and improve the uptake of modern period products. Period Products is an umbrella term that includes all products that are manufactured either locally or commercially for the specific purpose of absorbing menstrual blood, including sanitary pads, tampons, period pants and menstrual cups. The Indian government has been working to push the usage of sanitary pads since 2011 as a part of its Menstrual Hygiene Scheme (MHS) under the National Health Mission (NHM). Multiple state governments have also started various initiatives to improve the period product usage within their states [2] These initiatives have increased India’s sanitary pad usage from a mere 15% of menstruating women in 2010 [3] to 57% in 2015–16 [4] and further to 78% in 2019–21 [5] (see Fig 1).

One emerging concern is that public policy efforts have focussed only on improving the uptake of disposable sanitary pads and have ignored sustainable alternatives like reusable pads, reusable menstrual underwear, and menstrual cups [6, 7]. Disposables need monthly purchases and require disposal of large amounts of plastic which makes them inherently financially and environmentally unsustainable. Moreover, the recent pandemic exposed the vulnerabilities of the global distributional supply chains for disposables, calling into question their reliability [8].

Heavy reliance on disposable period products means the incidence of period poverty remains high in India, especially among the urban poor. More than a decade after India’s menstrual health policy and associated programmes were initiated, evidence suggests that the incidence of period poverty in India continues to remain precarious, with a large proportion of users still reliant on government subsidies and hand-outs. Drawing on a nationally representative dataset, Babbar and Dev (2021) find that just 39% of the households reported spending on period products in January 2020, thus, highlighting the period poverty crisis in India before COVID-19 struck [9]. The situation further worsened during the pandemic as period product consumption further slumped by 16% (ibid.). This was compounded by a more than 50% fall in sanitary pad distribution as part of the MHS scheme due to the COVID-19 lockdown in 83 districts, across 11 states of India [10]. This evidence suggests that a mere focus on promoting use of disposable pads is not enough to tackle period poverty, which requires a wider level of commitment from government and policymakers, including ensuring the availability of a range of affordable and accessible period products [11].

Moreover, a growing population means that the increasing popularity of disposable pads has created a huge ecological burden in India. A study by The Ministry of Drinking Water and Sanitation, Government of India shows that girls and women use 12 billion pads annually [12] This is estimated to generate menstrual waste amounting to 9,400 tonnes per month or 1,12,800 tonnes annually [13]. Given the sizeable waste produced by disposables, it is crucial for public policy that we understand the factors that support the use of sustainable alternatives like menstrual cups.

Concerns around the financial and ecological burden of disposables have seen several innovations in the range of sustainable period products. While there are several sustainable product lines, the two main ones on offer are reusable cloth pads and the menstrual cup. The low lifecycle cost of these products and low ecological impact makes them a much cheaper alternative to disposables. Especially menstrual cups which are estimated to have less than 1.5% of the environmental impact of disposables at 10% of the cost [14]. A single cup can hold 10 to 38ml of blood and depending on the flow, it needs emptying between 4 to 12 hours [15]. A menstrual cup can last up to 10 years and costs between 200 to 2000 (INR), thus, making it one of the cheapest but environment-friendly options.

However, the usage of the menstrual cups remains low in India. According to a national health survey, just 0.3% of women aged 15–24 in India use menstrual cups [5]. One of the main reasons behind the low usage of menstrual cups is the requirement for vaginal insertion. A large proportion of the population believes that the use of menstrual cups will break the hymen of the girl and she will no longer remain a virgin [16]. For policymakers to alleviate period poverty and to contain the environmental burden of menstrual waste, it is essential to identify the correct levers to improve the uptake of modern period products and within that to promote the use of sustainable period products that can help tackle period poverty sustainably and resiliently.

In this study, we use a nationally representative dataset from India to examine two issues–first, we aim to understand the socio-demographic factors that support the use of any modern period products among girls and women and second, we take a closer look at those who use modern products to examine factors that promote disposable vs. sustainable menstrual choices. We are specifically interested in studying how the socio-demographic factors for the girls and women using disposable period products (mainly pads and tampons) differ from those using modern sustainable period products (menstrual cups). This is a significant question not only given the high incidence of period poverty in the country but also the increasing ecological burden of waste load generated due to the usage of disposable period products, including sanitary pads and tampons, and the need for a shift in consumer behaviour to more sustainable options.

Our paper makes multiple contributions to the growing strands of the literature on menstrual health. First, we contribute to the literature on modern-period product usage in the Global South [17–26]. Specifically, this study helps understand the socio-demographic differences in disposable vs. reusable period product usage among girls and women in India. These findings would be relevant to the other LMICs with similar socioeconomic and cultural contexts. Second, we contribute to the emerging literature on the issues related to gender and public health [10, 27–29]. Our findings are particularly significant from the perspective of informing governments and policymakers on the key areas of focus to improve the usage of reusable period products, thereby making menstrual health policies more sustainable and resilient.

## Menstrual health and hygiene management in India

Effective menstrual hygiene management is critical for women and girls to fully participate in society with comfort and dignity. One of the critical components for good menstrual hygiene is the appropriate use of a menstrual absorbent. These may include cloth, sanitary napkins, locally prepared napkins, menstrual cups, tampons, or a combination of these materials [5]. In countries with a higher prevalence of period poverty, a large proportion of girls and women use old clothes or rags to manage their menses. In India, over 50% of girls and women of menstrual age are said to use cloth [5]. Using old cloth and rags or non-usage of period products may result in negative health outcomes, such as urinary and sexual tract infections [17, 18]. In the case of cloth, evidence suggests that unhygienic use may result because of lack knowledge of the appropriate management of menstrual cloth and also because of traditional taboos surrounding menstruation which constrain women’s ability to dry cloth in direct sunlight [30].

Access to basic sanitation and hand-wash facilities are other crucial factors in managing the menstrual needs of the individuals who menstruate. Poor WASH infrastructure in schools has been proven to affect adolescent girls’ enrolment rates globally [31, 32] as it is one of the main factors in enabling the management of MHH needs [19, 20] In India, a significant proportion of the population suffers from a lack of basic WASH facilities. Two surveys conducted by RICE between 2014 and 2018 revealed that 44 percent of the Indian population defecated in the open [33] and 76th Round NSSO Report (2018) on drinking water, sanitation, and hygiene shows that 29 percent of the Indian population had no access to toilets. Additionally, around 27% of girls and women in low-income countries lack basic facilities to wash their hands [34]. Previous studies have also shown that poor management of menstrual needs may lead to higher levels of stress, social stigma, as well as feelings of shame and embarrassment [35, 36], which further infringes their right to basic public health, human rights [37].

One stream of evidence suggests that lack of information on menstruation, including knowledge of products and their appropriate use, is far too common and a serious policy concern. In a recently conducted meta-analysis that included 97,070 girls over 138 studies in India, it was found that around half of the adolescent girls attained menarche with little understanding of how to manage it [38]. This experience is made worse by the various myths and false beliefs about menstruation that are levied on them by their communities and families, such as prohibitions on taking a bath, using the kitchen, going to religious places, and other forms of discriminatory practices (such as untouchability and social isolation) by their family friends and close relatives [39, 40]. Such biases and behaviours that uphold myths may severely affect girls, including their absence from school while they are menstruating and ultimately dropping out of school [41].

Formal education is seen to makes little difference to women’s menstrual knowledge and beliefs [28, 42]. A cultural of silence surrounds menstruation makes it very difficult even for educated women to seek information about periods, including products and their appropriate use. While commercial marketing campaigns have ensured that disposable sanitary pads are widely known, awareness of sustainable products like menstrual cups is low due to tighter profit margins and low-marketing budgets. Awareness of menstrual cups (and other reusable products) is negligible not only in developing countries like India [43] but also in developed countries like the UK [44]. In such a situation, women are effectively denied the agency to choose the menstrual product that enables them to manage their menses optimally, both for themselves and their environs as fits their requirements (see [6, 43, 45]).

As discussed earlier, menstrual cups offer a sustainable, cost-effective, and resilient menstrual hygiene alternative to disposable pads. Despite their appeal, menstrual cups have received little traction in the Global South mainly due to the prevalence of patriarchal taboos against vaginal insertion that prevail in many cultures [16]. However, evidence suggest that menstrual cups have a high acceptability rate among women who have used them. Given that their take up started in the US in 1930, many of the studies are based in high-income countries as they were found be to more comfortable, have lower odds of leakage, had less odour, and required to changed less frequently as compared to other disposable period products, including sanitary pads and tampons [46–48]. Multiple studies from low-income countries have also shown acceptance of menstrual cups [29, 49–51]. A randomised-control trial (RCT) study conducted on 124 girls and women in South Africa showed that participants preferred using menstrual cups for comfort, quality, menstrual blood collection, appearance, and preference [49]. Two similar RCTs were conducted in Kenya and Nepal which showed high adoption of menstrual cups, however, acceptance came only with the provisioning of peer support and mentorship [50, 51]. These results were further confirmed in a meta-analysis conducted on 43 studies globally which showed that menstrual cups are a safe option to manage menstrual needs [52].

Menstrual cups certainly seem to have the potential to alleviate period poverty sustainably and resiliently, but it may require community and peer support [52]. It is also imperative to understand what factors determine their uptake and usage locally that may provide useful insights from a policy perspective.

In recently conducted systematic reviews, it was found that most studies have attempted to comprehend various facets of schoolgirls’ menstrual practices [36, 38]. These studies, however, fall short of capturing the socio-demographic differences among the individuals who use sustainable (menstrual cups) vs. disposable (sanitary napkins and tampons) period products [5]. In this study, we attempt to understand the socio-demographic differences among the individuals who use these different period products. Without the aid of marketing campaigns and public messaging, how do women find out about sustainable period products and decide to use them, what other socio-economic factors drive up the use of these products?

## Methodology

### Data

The study utilized the most recent nationally representative, large-scale, publicly available Indian version of the Demographic and Health Survey (DHS), also known as the National Family Health Survey (NFHS-5), conducted in 2019–21. The International Institute for Population Sciences, Mumbai, administers this survey in collaboration with the Ministry of Health and Family Welfare. The NFHS-5 gathers information using two questionnaires: a) household questionnaires, which collect data from all household members, and b) women’s questionnaires, which gather information from women in the households on their socio-demographic characteristics, sexual and reproductive health, including menstrual cycle, among other factors. The NFHS-5 data collection occurred in two phases. Phase I was conducted from June 2019 to January 2020 in 17 states and five union territories, while Phase II was conducted from January 2020 to April 2021 in 11 states and three union territories. This included data from 636,699 households with 724, 115 females, and 101,839 males [5] For menstruation-related questions, the NFHS-5 provides data for 241,112 women aged 15 to 24 years. This constitutes the sample used in this study.

### Dependent variable

We construct the dependent variables for this study by using the response options for the following question from the NFHS-5 questionnaire: “Women use different methods of protection during their menstrual period to prevent blood stains from becoming evident. What do you use for protection, if anything?” There were seven response options (1 = Cloth, 2 = Locally Prepared Napkins, 3 = Sanitary Napkins, 4 = Tampons, 5 = Menstrual Cups, 6 = Nothing, 7 = Other). In the first part of our enquiry, where we examine the socio-demographic factors that influence individuals to use any period product to manage their menstrual needs. Here, our dependent variable is ordered and takes the value 0 if respondents report using Nothing or Other (responses 6 and 7); takes value 1 if they report using traditional cloth (response 1), and value 2 if they report using any modern menstrual product (responses 2 to 5). We combine the two categories of responses as using Nothing and using Others (which includes materials like rags, hay, ash, etc) to manage menstrual needs as both are considered unhygienic and the least preferred alternatives. Note that we distinguish between the use of modern menstrual products and traditional cloth due to the reasons mentioned earlier which may render the use of cloth unhygienic in a country like India.

Our second research question aims to examine the socio-demographic determinants of individuals using a modern sustainable period product i.e., menstrual cups vs. disposable options like sanitary pad and tampons. Here we consider only girls and women who have reported using modern period products. We examine the socio-demographic differences between women using menstrual cups vs. disposable products (pads and tampons). For this specification, the dependent variable is binary and takes the value 0 if the participant selected a modern disposable product (responses 2, 3, and 4) and takes the value 1 if the participant reports using menstrual cups (response 5). Next, we examine more closely some of the interesting key determinants for the use of sustainable products.

### Independent variables

Several individual and household-level socio-demographic variables were used in this study. Individual level variables include Age at Menarche (Less than 12, 12 to 15 years, >15 years), Education, Marital Status (Never in the union, Married, Other), and Frequency of reading the newspaper, listening to radio, and watching television (Not at all, less than once a week, at least once every week). The frequency of reading the newspaper, listening to the radio, and watching television were merged to create a new variable mass media exposure. It takes the value 0 if a respondent does not have any exposure to any of the media. Similarly, it takes the value 1 and 2, if the respondent has exposure to these media items less than once a week and at least once a week, respectively. Household-level variables include Wealth Index (Poor, Middle, Rich), Water and Sanitation Facilities (Hygienic, Unhygienic), Social Groups (Scheduled Caste, Scheduled Tribe, Other Backward Classes, None/Don’t Know), Religion (Hindu, Muslim, Others), and Place of Residence (Urban, Rural). Please note that water and sanitation facilities combine three variables: toilet facility, source of drinking water, and time to get drinking water. Firstly, we created three distinct binary variables to represent each component. The "Toilet facility at home" variable is assigned a value of "1" if the toilet facility falls under any of the following categories: a) flush—to piped sewer system; b) flush—to septic tank; c) flush—to pit latrine; d) flush—don’t know where; e) pit latrine—ventilated improved pit (VIP); f) pit latrine—with slab; g) composting toilet; otherwise, it is assigned a value of "0." Similarly, the "Source of drinking water" variable is given a value of "1" if the drinking water source is categorized as any of the following: a) piped into dwelling; b) piped to yard/plot; c) public tap/standpipe; d) piped to neighbor; e) tube well or borehole; f) protected well; g) protected spring; h) rainwater; i) tanker truck; j) cart with a small tank; k) bottled water; otherwise, it is assigned a value of "0." The "Time to get drinking water" variable is binary, with a value of "1" if obtaining drinking water takes 30 minutes or less or if the drinking water source is within the premises; otherwise, it is assigned a value of "0." To create the composite variable "Water and Sanitation facilities," we assigned a value of "1" only if all three individual binary variables (toilet facility, source of drinking water, and time to get drinking water) are equal to "1." Otherwise, "Water and Sanitation facilities" is given a value of "0." The choice of these variables was informed by the literature examining the socioeconomic and mass media factors that influence menstrual choices [17, 19, 20, 22, 24–26].

### Empirical strategy

Data analysis was performed in Stata Version 15.1. We use the ordinal logistic regression model to identify the various socio-demographic factors influencing the usage of period products by women to manage their menstrual needs. Ordinal logistic regression is employed when the dependent variable is ordinal (varyingly categorical, indicating that it falls into a set of ordered categories) and there are more than two categories.

Ordinal logistic regression is employed in our paper as our dependent variable takes three values in the ordered form, i.e., using nothing/others, traditional cloth, and modern period products. Women using nothing to manage their periods are considered as the least preferred outcome, followed by those using traditional cloth, followed by the most preferred outcome of using modern period products (also see, [5]). Managing menstruation without proper products is associated with increased health risks. Insufficient protection and hygiene during menstruation may contribute to a higher likelihood of infections and discomfort.

On the other hand, the use of traditional cloth represents a step towards better menstrual hygiene compared to using nothing. However, it is important to note that cloth usage alone may still have limitations in terms of absorbency, leakage protection, and overall comfort. While it provides a more hygienic alternative to inadequate options, it may not offer the same level of convenience and effectiveness as modern period products.

Therefore, our study emphasizes the significance of modern period products, such as sanitary napkins or tampons, as the most preferred outcome. These products offer improved hygiene, convenience, and efficient management of menstrual needs, potentially minimizing health risks and enhancing overall well-being.

By employing ordinal logistic regression, we aim to capture the ordered nature of these choices and examine the factors influencing women’s preferences for different menstrual management options. We choose ordered logit regression over multinomial regression as the latter does not preserve the ranking information in the dependent variable while returning the results, i.e., the contribution of each of our independent variables.

The estimated model is shown below:

$$logit(P\left(Y\leq j\right))=\beta_{j0}+\beta_{1}X_{1}+\beta_{2}X_{2}+\ldots +\beta_{n}X_{n}+\varepsilon_{j}$$

where P (Y ≤ j) is the cumulative probability that the response variable Y (period product usage) is less than or equal to the j^th^ category (0 = nothing/others, 1 = traditional cloth, and 2 = any modern period product), *β*_*1*,_ *β*_*2*,_ *β*_*n*_ are the coefficients of independent variables X_1_, X_2_ upto X_n_, and *ε*_*i*_ is the error term.

The second stage of our enquiry is to understand the socio-economic factors that influence the use of modern disposable period products, i.e., pads and tampons vs. reusable products, i.e., menstrual cups.

The estimated model is shown below:

$$logit\left(p\right)=log(\frac{p}{1-p})=\beta_{0}+\beta_{1}X_{1}+\beta_{2}X_{2}+\ldots +\beta_{n}X_{n}$$

where p is the probability of the binary response variable Y which has two categories (0 = nothing/others/traditional cloth, and 1 = any modern period product), *β*_*1*,_ *β*_*2*,_ *β*_*n*_ are the coefficients of independent variables X_1_, X_2_ upto X_n_. In other words, we compare menstrual cup users against the users of any modern disposable menstrual product (sanitary napkins, locally prepared sanitary napkins, tampons) to manage their periods. In this specification, the dependent variable is binary and hence, binary logit regression was used to produce the estimates for the second set of results.

## Results

Table 1 presents the descriptive statistics for the study variables. The first column of the table shows that the majority of women participating in the NFHS-5 survey reside in the rural area (71%), and belong to the Hindu religion (80%), and other backward classes (45%). Around 87% of women in our sample have completed secondary education or more. A large proportion of the women are unmarried (64%). Almost 66% women in our sample use a hygienic water and sanitation facility and 58% of women have maximum exposure to mass media.

**Table 1:** Respondent’s characteristics and prevalence of period product usage by background characteristics of women aged 15–24, NFHS-5 (2019–21).

Respondent Characteristics	Overall Sample	Respondents reporting using any modern period product	p-value
Variables	Frequency (Percentage)	Prevalence (%)	
*Individual Level*			
Age at Menarche			
Less than 12 years	40909 (16.96)	32083 (78.43)	<0.01
12 to 15 years	189222 (78.46)	146623 (77.49)	
15 years and above	11048 (4.58)	7776 (76.90)	
Education			
No Education	15833 (6.51)	6876 (43.43)	<0.01
Primary	15028 (6.23)	8003 (53.56)	
Secondary	164574 (68.24)	129200 (78.86)	
Higher	45866 (19.02)	42481 (92.74)	
Marital Status			
Married	86702 (35.95)	62223 (71.91)	<0.01
Never Married	153228 (63.53)	123445 (80.97)	
Other	1248 (0.52)	814 (65.28)	
Mass media Exposure			
No	47661 (19.76)	25768 (54.39)	<0.01
Partial	54486 (22.59)	41590 (76.64)	
Full	139032 (57.65)	119125 (85.96)	
*Household Level*			
Wealth Index			
Poor	101142 (41.94)	63092 (62.75)	<0.01
Middle	50656 (21.01)	41599 (82.12)	
Rich	89375 (37.06)	81947 (91.85)	
Water and Sanitation Facility			
Unhygienic	83009 (33.58)	54726 (66.25)	<0.01
Hygienic	158171 (66.42)	132204 (81.79)	
Social Category			
General	56452 (19.68)	47233 (83.67)	<0.01
Scheduled Caste	55274 (24.04)	42411 (76.73)	
Scheduled Tribe	23191 (10.09)	15223 (65.64)	
Other Backward Classes	104663 (45.52)	81313 (77.69)	
Don’t Know/Unknown	1532 (0.67)	1026 (63.48)	
Religion			
Hindu	193622 (80.31)	150347 (77.65)	<0.01
Muslim	36757 (15.24)	27454 (74.69)	
Others	10733 (4.45)	9375 (87.35)	
Place of Residence			
Urban	70941 (29.42)	63549 (89.58)	<0.01
Rural	170171 (70.58)	123612 (72.64)	

The second column of the table reports the prevalence of modern period product usage reported by the girls and women in our sample. It shows that those who attained menarche between 12 to 15 years had a significantly higher period product usage than those who attain menarche aged 15 and over. Notably, period product usage is higher among unmarried women (80%), as compared to married women (70%). The period product usage also increases significantly with the increase in education. Around 43% of women with no education use period products, as compared to 93% of women with higher education. Individuals’ exposure to mass media seems to matter for period product usage. It increases from 54% for women with no mass media exposure to 86% for women with full mass media exposure. At the individual level, these summary statistics point to the huge gap in the usage of modern-period products created due to lower education and poor mass media exposure.

Examining household-level attributes, we find that household wealth, social group, and location matter for the usage of modern period products. Usage increases with the increase in wealth. In the poorest wealth strata, the usage of sanitary items is around 63%, whereas in the middle and richest wealth strata, the usage is as high as 82% and 92% respectively. In keeping with this result, households with hygienic Water and Sanitation facilities (82%) had considerably higher period product usage as compared to those who do not use hygienic Water and Sanitation facilities (66%). In general agreement with the trend, usage is observed to be considerably lower among the women belonging to the Scheduled Tribe (66%) and Scheduled Caste (77%) as compared to those from the General category (84%). Among the religious groups, the period product usage is lowest amongst Muslims (75%) followed by Hindus (78%) and other religious groups (87%). We also find that a significantly higher proportion of women surveyed in urban areas use modern period products than women in rural areas.

Col (1)-(2) of Table 2 displays the marginal conditional effects from the ordinal logit regression model for the respondent’s using cloth and all modern period products, as compared to those who use nothing/others. Women using cloth were negatively associated with the highest level of education as compared to those with no education (Clothes: β = -0.2635, p<0.01). In contrast, women using period products increased with the level of education compared to those with no education (β = 0.2681, p<0.01). Usage of Cloth for menstrual protection was negatively associated with mass media exposure, wealth index, and Water and Sanitation facility. In contrast, those using period products for menstrual protection were positively associated with these factors.

**Table 2:** Odds ratio for girls and women using different methods for menstrual protection.

Variables	Ordinal Logit Regression		Logit Regression
	(1)	(2)	(3)
	Cloth	Period Products	Period Products
*Individual Variables*			
Age at Menarche			
Less than 12^®^			
12 to 15 years	-0.0065**	-0.0076**	1.04**
15 years or older	-0.0100**	-0.0174**	1.11***
Education Level of Respondents			
No Education^®^			
Primary	-0.0412***	0.0422***	1.25***
Secondary	-0.1772***	0.1806***	2.61***
Higher	-0.2635***	0.2681***	4.91***
Marriage			
Single ^®^			
Married	0.0225***	-0.0228***	0.82**
Other	0.0786***	-0.0798***	0.63***
Mass media Exposure			
None^®^			
Partial	-0.0866***	0.0880***	1.65***
Full	-0.1173***	0.1192***	1.92***
*Household Variables*			
Wealth Index			
Poorest ^®^			
Middle	-0.0959***	0.0975***	1.62***
Richest	-0.1585***	0.1609***	2.61***
Water and Sanitation Facility			
Unhygienic^®^			
Hygienic	-0.0297***	0.0302***	1.09***
Social Category			
General^®^			
Scheduled Caste	0.0161***	-0.0163***	0.85***
Scheduled Tribe	0.0584***	-0.0592***	0.74***
Other Backward Classes	0.0304***	-0.0309***	0.86***
Others	0.0788***	-0.801***	0.58***
Religion			
Hindu^®^			
Muslim	0.0419***	-0.0425***	0.83***
Others	-0.0657***	0.0667***	1.21***
Place of Residence			
Urban^®^			
Rural	0.0577***	-0.0586***	0.71***
Observations	228,796	228,796	228,796

Col (1)-(2) displays the marginal effects from the ordinal logit regression of using cloth and modern period products, respectively, as compared to nothing/others. Col (3) displays the odds ratio from the binary logit regression from using the period products as compared to those using unhygienic ways of managing menstrual needs, including clothes, nothing, and others (Anand et al., 2015). ^®^ denotes the reference category.

Col (3) of Table 2 displays the odds ratio from the binary logit regression model for the respondents using modern period products as compared to those using unhygienic ways of managing menstrual needs i.e., clothes, nothing, and others. Women using modern period products were positively associated with the highest level of education as compared to those with no education (OR = 4.91, p<0.01). The usage of modern period products increases with increased exposure of mass media (Partial Exposure: OR = 1.65, p<0.01; Full Exposure: OR = 1.92, p<0.01). Usage of modern period products increases with the increase in the income levels of the families. Women coming from middle (OR = 1.62, p<0.01) and richer backgrounds (OR = 2.61, p<0.01) have higher period product usage as compared to those coming from poorer background. Compared with women from general caste, those from scheduled caste, scheduled tribe, and other backward classes had lower usage of modern period products (ST: OR = 0.74, p<0.01; OBC: OR = 0.86, p<0.01). Modern period product usage was lower among the women in the rural areas as compared to those in the urban areas (OR = 0.71, p<0.01).

Table 3 presents the descriptive statistics of the respondents across various types of period products categorised as Sustainable Products and Disposable Products. Only 0.3% of women in India aged 15–24 use reusable period products, i.e., menstrual cups. In contrast, 77% of them use disposable period products i.e., sanitary napkins and tampons. Like before, we find that women with highest education levels and full media exposure are significantly more likely to use reusable and disposable period products as compared to the women with lower levels of education and lack of exposure to media. We also found similar results for women from rich wealth quantile, with hygienic Water and Sanitation facility and general caste categories. Again, we find that significantly higher proportion of women living in urban areas use reusable or disposable period products when compared to their rural counterparts.

**Table 3:** Descriptive statistics of the respondents using different period products.

Variables	Disposable Products (sanitary pads and tampons)	Sustainable Products (menstrual cups)
Period Product Usage	186610 (77.12)	730 (0.3)
*Individual Variables*		
Age at Menarche		
Less than 12 years	32096 (78.19)	156 (0.38)
12 to 15 years	146735 (77.29)	547 (0.29)
15 years and above	7780 (70.18)	26 (0.24)
Education		
No Education	6811 (43.21)	14 (0.09)
Primary	7997 (53.05)	37 (0.25)
Secondary	129282 (78.29)	498 (0.30)
Higher	42518 (92.39)	180 (0.39)
Marital Status		
Married	62284 (71.60)	203 (0.23)
Never Married	123512 (80.34)	524 (0.34)
Other	815 (65.05)	2 (0.17)
Mass media Exposure		
No	25796 (53.94)	70 (0.15)
Partial	41592 (76.08)	187 (0.34)
Full	119223 (85.47)	472 (0.34)
*Household Variables*		
Wealth Index		
Poor	63119 (62.20)	236 (0.23)
Middle	41483 (81.61)	125 (0.25)
Rich	82009 (91.45)	369 (0.41)
Water and Sanitation Facility		
Unhygienic	54551 (66.85)	249 (0.35)
Hygienic	128557 (82.31)	562 (0.41)
Social Category		
General	38442 (84.64)	139 (0.31)
Scheduled Caste	42306 (76.26)	144 (0.26)
Scheduled Tribe	15168 (65.14)	74 (0.32)
Other Backward Classes	81065 (77.19)	321 (0.31)
Don’t Know/Unknown	964 (62.43)	6 (0.38)
Religion		
Hindu	149910 (77.14)	559 (0.29)
Muslim	27345 (74.14)	127 (0.34)
Others	9356 (86.93)	44 (0.41)
Place of Residence		
Urban	63334 (89.08)	344 (0.48)
Rural	123277 (72.14)	386 (0.23)

Table 4 displays the odd ratios from the binary logit regression for the women using the reusable period products i.e., menstrual cups compared to those using disposable period products (sanitary napkins, locally prepared sanitary napkins, tampons) to manage menstrual needs. Compared to other methods of menstrual protection, menstrual cup usage increases with the increase in the education level of the respondents (Higher Education: OR = 1.68, p<0.05). Menstrual cup usage was lower among the women in rural areas as compared to those in urban areas (OR = 0.73, p<0.01). Menstrual cup usage was positively associated with mass media exposure, both partial exposure (SC: OR = 1.38, p<0.05) and maximum exposure (SC: β = 1.34, p<0.01). Compared with women from the general caste, those from scheduled caste, scheduled tribe, and other backward classes had higher chances of menstrual cup usage (SC: OR = 1.57, p<0.01; ST: OR = 1.36, p<0.05; OBC: OR = 1.42, p<0.01).

**Table 4:** Results from binary logit regression (odds ratio) for women by background characteristics using menstrual cups vs other products to manage their menstrual needs.

Variables	Menstrual Cups vs Disposable Period Products	
	(1)	(2)
*Individual Variables*		
Age at Menarche		
Less than 12^®^		
12 to 15 years	0.89	1.11
15 years or older	0.79	0.94
Education Level of Respondents		
No Education^®^		
Primary	1.87**	1.82**
Secondary	1.57*	1.39
Higher	1.86**	1.68*
Marriage		
Single ^®^		
Married	0.84**	0.90
Other	0.59	0.70
Mass media Exposure		
None^®^		
Partial	1.42**	1.38**
Full	1.30**	1.34**
*Household Variables*		
Wealth Index		
Poorest^®^		
Middle	0.84	0.92
Richest	0.98	1.14
Water and Sanitation Facility		
Unhygienic ^®^		
Hygienic	0.82	0.96
Social Category		
General^®^		
Scheduled Caste	1.25*	1.57**
Scheduled Tribe	1.25	1.36*
Other Backward Classes	1.10	1.42**
Don’t Know	1.74	2.31*
Religion		
Hindu^®^		
Muslim	1.79***	1.11
Others	0.60**	0.85
Place of Residence		
Urban^®^		
Rural	0.83**	0.73**
State FE	N	Y
Observations	175,219	171,663

Note:

* p<0.1,

**p<0.05,

*** p<0.01.

^®^ denotes the reference category.

From the perspective of public policy, one important factor that emerges in these results as favouring the use of sustainable period products is the exposure to mass media. Given this, we examine the influence of mass media exposure by considering its various components more closely. Exposure to mass media constitutes the number of times an individual engages with any of the three mass media outlets: newspaper, radio, and television. Table 5 examines the influence of each of these separately on the usage of menstrual cups as compared to other methods to manage periods. Notably, we find that women reading newspaper or listening to radio at least once a week tend to have higher menstrual cup usage as compared to the women not reading newspaper or listening to radio.

**Table 5:** Results from binary logit regression by mass media factors for women using menstrual cups vs nothing/others to manage their menstrual needs.

Variables	Menstrual Cups vs Disposable PP	
	(1)	(2)
Reading Newspaper		
Not at all ^®^		
Less than once a week	1.13	1.22**
At least once a week	1.42**	1.58***
Listening to Radio		
Not at all^®^		
Less than once a week	1.98***	1.73***
At least once a week	3.14***	2.61***
Watching Television		
Not at all^®^		
Less than once a week	1.20	1.14
At least once a week	0.95	0.97
State FE	N	Y
Observations	175219	171663

Note:

* p<0.1,

**p<0.05,

*** p<0.01.

^®^ denotes the reference category.

### Robustness checks

We ran multiple robustness checks to confirm the main results. One of the main checks is including theoretically relevant control variables that we could not consider in our main regressions due to the limitation that these are collected only for a considerably smaller sub-sample of married women in the age group of 15–24. We include three such variables: women’s mobility; mobile phone ownership and wife-beating attitudes.

Women’s mobility or ‘freedom of movement’ is likely to determine whether they can access a period product of their own choice. To account for it, we include a variable that controls for the mobility of the women in our sample. Freedom of movement is measured using three questions from the NFHS questionnaire, i.e., “Are you usually allowed to go to the following places (a) market (b) health facility (c) place outside the community” with options (0 = Not at all, 1 = With someone else only, 3 = Alone). Presented in Col (1) of Table 6, the result suggests that women whose mobility is lower have lower odds of using menstrual cups (OR = 0.93, p<0.10).

**Table 6:** Robustness checks.

Variables	(1)	(2)	(3)	(4)
	Mobility	Mobile Phones	Wife-beating attitudes	All
*Additional Controls*				
Freedom of Movement	0.93*			0.93
Wife-beating attitudes			1.13	1.13
Usage of Mobile Phone				
No				
Yes		1.80**		1.12
*Individual Variables*				
Age at Menarche				
Less than 12^®^				
12 to 15 years	0.86	0.86	1.27	1.25
15 years or older	0.60	0.60	0.90	0.89
Education Level of Respondents				
No Education^®^				
Primary	0.92	0.93	1.90	1.93
Secondary	1.34	1.33	2.99	3.00
Higher	1.37	1.37	6.06*	6.27*
Mass media Exposure				
None^®^				
Partial	1.20	1.21	0.95	0.96
Full	0.99	0.99	0.78	0.80
*Household Variables*				
Wealth Index				
Poorest^®^				
Middle	1.19	1.18	1.38	1.33
Richest	1.96*	1.96**	0.94	0.90
Water and Sanitation Facility				
Unhygienic^®^				
Hygienic	0.99	0.99	0.61	0. 61
Social Category				
General^®^				
Scheduled Caste	3.81**	3.81**	10.75**	11. 06**
Scheduled Tribe	2.49*	2.49*	3.09	3.20
Other Backward Classes	2.99**	2.99**	6.64*	6.79*
Religion				
Hindu^®^				
Muslim	1.95*	1.95**	5.95**	5.88**
Others	0.20**	0.20**	0 (empty)	0 (empty)
Place of Residence				
Urban^®^				
Rural	1.90**	1.90**	2.72	2.74
Constant	0.002***	0.002***	0.0002***	0.0004***
Observations	27,942	27,942	4,246	4,246

Notes: Marriage is omitted due to high multicollinearity.

* p<0.1,

**p<0.05,

*** p<0.01.

^®^ denotes the reference category.

Another empowering variable that may influence women’s period products usage is mobile phone. There are multiple applications available online which can empower women with the information regarding the usage of various period products and best practices to manage their menstrual needs. To accommodate this, we control for the mobile phone usage, as shown in Col (2) of Table 6. Our results show that women using mobile phones have higher odds of using menstrual cups (OR = 1.80, p<0.05).

Wife-beating attitudes could also influence period product usage, either via their influence on women’s mobility or via traditional beliefs around using period products. We measure wife-beating attitudes using the NFHS questions on attitudes around domestic abuse. These questions include, is a husband/partner justified in hitting or beating his wife in the following situations, i.e., “If she goes out without telling him?” “If she refuses to have sex with him,” “If she doesn’t cook food properly,” “If he suspects her of being unfaithful,” “If she shows disrespect for in-laws.” The responses were dichotomous: 0 = No or Don’t Know, 1 = Yes. We created a new binary variable ‘*wife-beating attitudes*’, which takes the value 1 if women justify even one of these items. Col (3) of Table 6 displays the results for the women using menstrual cups after controlling for their wife-beating attitudes. However, there was no significant association of wife-beating attitudes with the women using menstrual cups.

## Discussion

A combination of commercial interests and public policy over the last two decades have resulted in a steep rise in the take-up of disposable sanitary pad in several LMICs. A case in point is India, where the use of period products has experienced a sharp increase from around 12% in 2010 to 78% by 2021. This increase has been accompanied by worries over both the environmental and financial sustainability of promoting the use of disposables. The recent pandemic also exposed the supply chain vulnerabilities of such products given the heavy reliance on global distributional channels. Concerns around sustainability have seen several innovations in the range of sustainable period products available to women to manage their menses. Menstrual cups have been the most prominent of these, but there are also other products like modern-grade reusable cloth pads, period panties and so on. Several of these products have low lifetime costs and represent low environmental burden as compared to disposable pads. Understanding what factors promote the uptake of reusable period products is critical to shaping policies that can promote sustainable, affordable, and resilient menstrual hygiene for everyone.

In this study, we examine the socio-demographic factors that determine sustainable/reusable period product usage in India. Most of the socio-demographics we consider in this study are significantly associated with the use of modern period product. This includes individual-level variables like age at menarche, education level, mass media exposure, and household-level ones like social category, religion, wealth index, Water and Sanitation facility, and household location. However, for reusable period products, only a bunch of these socio-demographic factors i.e., education, mass media exposure (newspapers and radio), social category, and household location.

Our findings show that the education level of the respondents matters for the use of sustainable period products. This result suggests that education may support the take up of menstrual cups both directly by improving their awareness and knowledge of menstrual cups and indirectly by helping women overcome the taboos surrounding vaginal insertion that may hinder the take up of menstrual cups. There are several studies that support the argument that education supports women make informed menstrual choices. For instance, studies have shown that women with higher education are more likely to choose hygienic practices to manage their menstrual needs [20, 40]. Education also helps in demystifying the taboos and myths around menstruation and empowers women to move freely, make autonomous decisions, and have financial independence [21, 22].

Another important factor contributing to the use of sustainable period products is women’s exposure to mass media improves the usage of menstrual cup. Note that while our results on exposure to radio are the strongest, our results on exposure to television are the weakest. There are several reasons that make radio an effective policy tool. It is a medium that can reach a large, under-literate population and communicate messages in regional languages. Radio is also unique as it is a medium that is more private than other media outlets. For instance, it is common for women from middle and lower-middle classes to listen to daytime radio programmes while they are alone cooking and cleaning, while men and children are out of the household. It is in recognition of this that radio programmes that discuss women’s reproductive health issues, tackling more difficult-to-access and culturally taboo subjects are transmitted at times that are convenient for women to listen in on their own [21]. Radios are also typically mobile and can be carried into private spaces like bathrooms. Discussions of taboo subjects are less acceptable on television as this is typically kept in the main living area of the household and remains fixed and watching television largely remains an evening or holiday family activity. Radios are also far cheaper than televisions making their ownership far wider, including in rural areas. These reasons have made radio an especially powerful tool in the past for communicating public health messages to the masses and government nodal ministries like Ministry of Health and Family Welfare have conducted programs to improve the reproductive and health issues [53]. Radio has also been used in the past in programmes like “*18 to 82*” to help promote menstrual awareness. Programme “*18 to 82*” bridge the gap between the 18% individuals who use sanitary pads to manage their menstrual needs and 82% who do not use a hygienic method to manage their menstrual needs. Some radio programmes already mention reusable period products, and this could be further enhanced to help with awareness.

Our result also shows that women from disadvantaged social groups (scheduled caste, scheduled tribe, and other backward classes) have a relatively greater probability of using menstrual cups when compared to advantaged social groups. This is a significant result, especially when we consider that women from disadvantaged social groups are relatively less likely to use modern period product overall when compared to the advantaged social groups. There may be two reasons for this. Women from these groups are typically also likely to be financially disadvantaged and hence likely to find the price of disposables a hurdle to the adoption of modern period products. However, women from the same disadvantaged social groups are also likely to be open to the idea of using a cost effective reusable modern period product as they are less likely to be inhibited by taboos around vaginal insertion. This result really focuses attention on the potential of menstrual cups to mitigate period poverty among disadvantaged social groups that constitute over 68% of India’s population [54].

We also find that women in the rural area have lower period product usage as compared to those in urban areas. There have been multiple government schemes conducted in the past to improve the period product usage in rural areas [2]. So far, most of these schemes have focused on improving the hard infrastructure, i.e., provision of subsidised or free disposable pads and do not include the complementary soft infrastructure, including behavioural interventions to target the taboos and myths around menstruation and knowledge around the range of period products and the correct usage. Another potential factor driving the poor usage in the rural areas could be the limited mobility of the women, and even those who could go out may not be able to buy the period products from the male shopkeepers or chemist due to the existing taboos and myths around menstruation in their communities or due to the lack of availability of such products [55]. Our results are backed by the previous studies which also show that the usage of period products is lower in the rural areas, as compared to the urban areas [18, 19, 26].

From a public policy perspective, our results shine a beacon on the idea that women who have the awareness and information about sustainable alternatives to disposal pads are from communities where traditional taboos are likely to be more relaxed and are living in locations where they are relatively free to move around and access such products are likely to assert their choice of period products that are both ecologically and financially sustainable. These choices are also likely to be much more resilient to the supply chain vulnerabilities that affect disposable products during COVID-19 lockdowns, as sustainable alternatives such as menstrual cups and reusable cloth pads are not reliant on external production and distribution networks, making them more readily available and accessible to women even in situations of disrupted supply chains. Our findings call for a shift in public policy perspective from popularisation of single product category to a focus on addressing barriers to information and awareness among women on the available range of period products, especially in rural areas and women from disadvantaged social groups, where education and income levels are lower via different mass media channels to improve reusable period product usage. It is also important to improve the availability of such products locally, so women with restricted mobility have better odds of accessing these products.

Several NGOs working on period poverty have recognised the potential of improving awareness of sustainable period alternatives among women as an effective strategy for menstrual health management [45, 56]. One example is the program implemented by the WSSCC, which uses a collapsible tent to create a safe space for girls and women to talk about menstrual health and introduce them to multiple types of menstrual products including compostable pads and menstrual cups [57]. Their report suggests that using a basket of products is a good tool for starting a conversation on menstrual health. The Sikun Relief Foundation (SRF) used a similar approach to increase awareness about menstrual hygiene and waste management in flood-prone regions of Assam [58]. Following the success of these initiatives, the Government of India introduced a menstrual training manual that includes information on all products, including their pros and cons, for its community health workers that serve rural communities [59].

Other organizations, such as the AKDN, Sukhibhava, HasiruDala, and Tata Trusts, go a step further and offer a basket of products to the communities they work in. They report higher awareness among girls and women regarding various aspects of menstruation and more appropriate use of menstrual hygiene products [60]. Based on interest from the community on user preference as well as affordability, the AKDN programme offered training for making reusable cloth pads and appropriate use of cloth pad as a sustainable option. By offering alternative products, these programs have explored ways to manage menstrual waste more effectively by leveraging existing practices and the community’s behaviour. Reports from these initiatives suggest that informed choice can be a powerful tool for equipping women with the information and products they need for managing their menstruation safely and sustainably.

## Limitations

This work has several limitations typical of studies based on national-level secondary data. The first of these is that the take-up of menstrual cups in India is low so far. Just 0.3% of our sample reported using menstrual cups. This means we have an extremely skewed sample for which other estimation strategies like propensity score matching would have been more appropriate. However, this would have reduced our sample significantly and defeated the purpose of exploring the socio-economic differences between girls and women who use disposable vs. sustainable modern period products. Secondary data also means that we do not have the advantage of exploring the primary results any further and are left to hypothesise about the underlying mechanisms that lead girls and women to choose sustainable menstrual products. Despite this, we have ensured that our hypothesis is verified (see Table 5) and its robustness tested (see Table 6), providing further confidence in our results.

Third, our study sample is limited to girls and women aged 15–24 as questions around period product usage were asked only to girls and women within this age group. Women between the ages of 15 to 24 make up around 12% of India’s population [61] and while it is important to understand their menstrual management practices, data limitations mean that we are unable to comment on menstrual choices made by older women.

Fourth, our study limits the data only to girls and women and does not provide any information on trans and non-binary individuals who menstruate [62].

## Concluding comments

Multiple countries across the world provide free period products as a part of their menstrual health policies. In most of these countries, there is a heavy reliance on the usage of disposable period products, more specifically, on sanitary pads. Sanitary pads generate a large amount of wastage and slowly and steadily, the menstrual hygiene narrative is moving towards sustainable periods via the usage of menstrual cups. However, to the best of our knowledge, no existing studies have examined the socioeconomic determinants of sustainable period product use vs. disposable products using a nationally representative database.

This study investigates this issue for a prominent LMIC–India, which is home to 20% of the world’s individuals who menstruate and thus makes an important contribution to knowledge in this area. Our analysis suggests that despite current low usage, menstrual cups hold the potential to mitigate period poverty sustainably and resiliently for a developing country with a large population. Our results show that despite reporting a relatively low usage of modern period products overall, socially disadvantaged groups report a relatively higher uptake of menstrual cups. This may be because costs associated with disposable products may be a bigger concern to women in these groups and taboos around vaginal insertion may be less prominent among these groups. Our other prominent result is that knowledge transmission may be key to the promotion of sustainable period alternatives. There is special potential for mediums like radio to be used to reach low-literacy communities in large numbers to address taboos regarding the use of menstrual cups and their association with virginity and to promote their usage. Similar radio programmes have been used in the past to transmit health messages to the masses with good results. As a public policy tool, this may have significant benefits in terms of improving sustainable menstrual health in India.
